# Multiple rapidly growing desmoid tumors that were difficult to distinguish from recurrence of rectal cancer

**DOI:** 10.1186/s12957-017-1248-7

**Published:** 2017-10-03

**Authors:** Koki Nakanishi, Dai Shida, Shunsuke Tsukamoto, Hiroki Ochiai, Junichi Mazaki, Hirokazu Taniguchi, Yukihide Kanemitsu

**Affiliations:** 1 Department of Colorectal Surgery, National Cancer Center Hospital, 5-1-1, Tsukiji, Chuo-ku, Tokyo, 1040045 Japan; 2 Pathology and Clinical Laboratory Division, National Cancer Center Hospital, 5-1-1, Tsukiji, Chuo-ku, Tokyo, 1040045 Japan

**Keywords:** Desmoid tumors, Mesenteric fibromatosis, Rectal cancer, Intra-abdominal, Cancer recurrence

## Abstract

**Background:**

Intra-abdominal desmoid tumors are usually slow growing and solitary, but multifocal desmoid tumors develop on rare occasions. Diagnosing desmoid tumors before histological examination of a surgical biopsy is often difficult. In particular, if a patient has a prior history of malignancy, it may be difficult to differentiate between these lesions and disease recurrence or metastasis.

**Case presentation:**

We present a rare case of multiple rapidly growing intra-abdominal desmoid tumors after surgical trauma, without familial adenomatous polyposis. A 51-year-old male underwent abdominal perineal resection with lateral lymph node dissection after neoadjuvant chemotherapy for lower rectal cancer. Follow-up computed tomography (CT), performed 6 months after primary surgery, showed a 20-mm solitary mass in the pelvic mesentery. Another CT scan, performed 3 months later, revealed that the mass had grown to 35 mm in size and that two new masses had formed. Based on imaging studies and his medical history, it was difficult to distinguish the desmoid tumors from recurrence of rectal cancer. Curative resection was chosen for therapeutic diagnosis. The pathological diagnosis was multiple mesenteric desmoid tumors.

**Conclusions:**

Desmoid tumors should not be excluded as a differential diagnosis for intra-abdominal masses after intra-abdominal surgery, even in cases of rapidly growing multiple masses.

## Background

A desmoid tumor is a monoclonal fibroblastic proliferation that arises from deep soft tissue and is characterized by infiltrative growth and a tendency toward local recurrence but an inability to metastasize [1]. Desmoid tumors are usually solitary, but multifocal desmoid tumors develop on rare occasions, comprise less than 5% of all desmoid tumor cases, and typically occur at the extremities in premenopausal women [2]. Intra-abdominal desmoid tumors can be sporadic or occur in association with familial adenomatous polyposis (FAP) [3]. Diagnosing desmoid tumors before histological examination of a surgical biopsy is often difficult. In particular, if a patient has a prior history of malignancy, it may be difficult to differentiate between these lesions and disease recurrence or metastasis [4–6]. Here we report a case of multiple rapidly growing intra-abdominal desmoid tumors which appeared 6 months postoperatively, which could not be differentiated from recurrence of rectal cancer.

## Case presentation

**Fig. 1:**
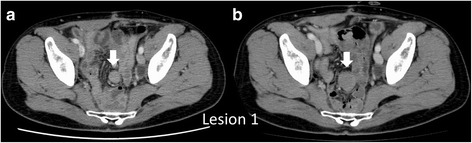
Contrast-enhanced computed tomography (CT) findings. **a** Abdominal CT shows a solitary tumor measuring 20 × 20 mm in size located at the mesentery of the small bowel inside the pelvis (arrow). **b** Three months later, tumor size increased to 35 × 30 mm (arrow)

**Fig. 2:**
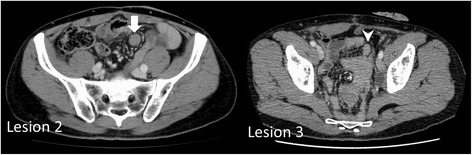
CT findings 3 months after previous CT. Abdominal CT shows two new solitary masses measuring 20 × 15 mm (arrow) and 15 × 10 mm (arrowhead) located at the mesentery of the small bowel

**Fig. 3:**
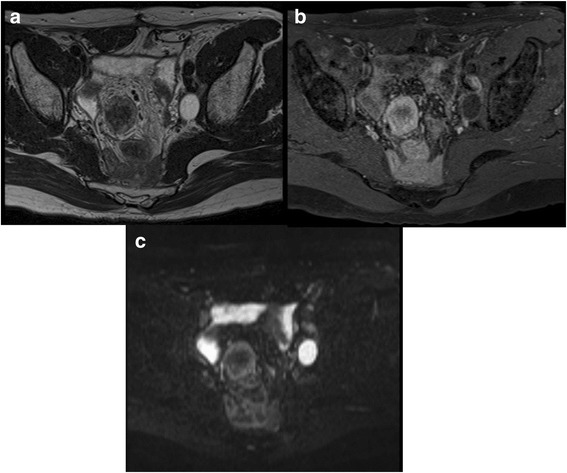
Magnetic resonance imaging (MRI) findings. **a** On a T2-weighted image, the central part is hypointense and the peripheral part is hyperintense. **b** On a gadolinium-enhanced image, the tumor is gradually enhanced. **c** On a diffusion-weighted image, the peripheral part is hyperintense

**Fig. 4:**
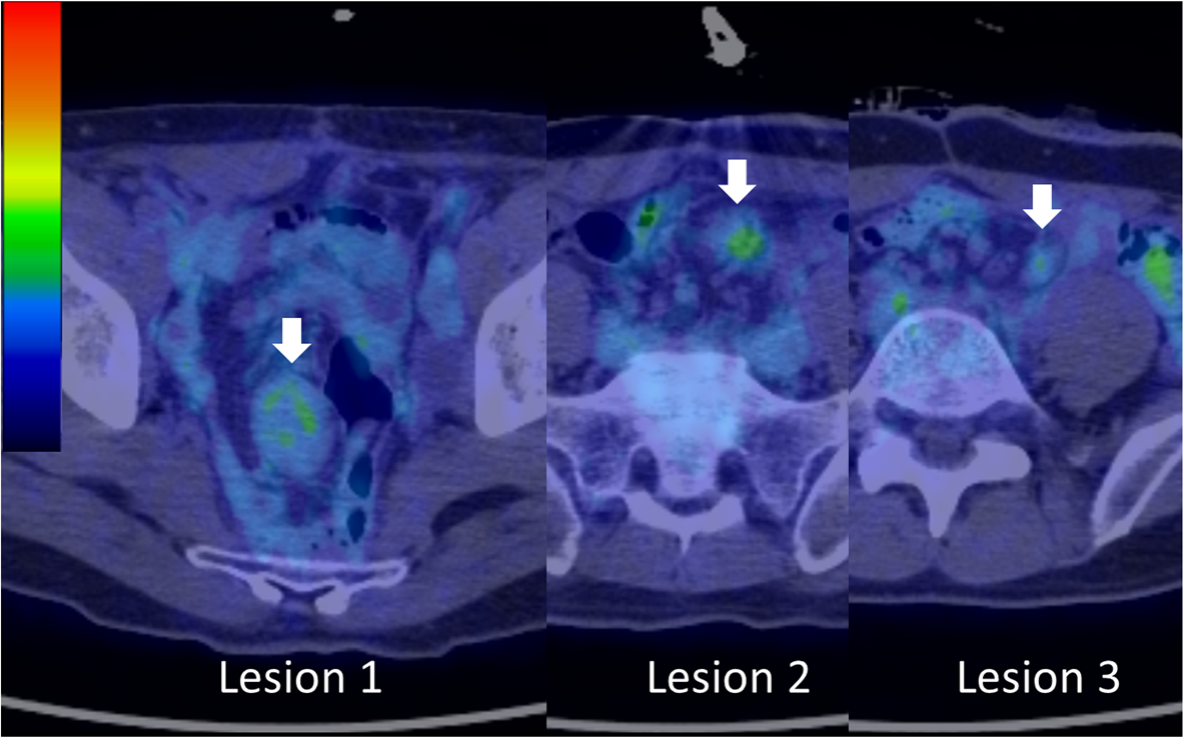
Positron emission tomography (PET-CT) findings. PET-CT shows increased fluorodeoxyglucose (FDG) uptake in all tumors

A 51-year-old male, who had received neoadjuvant chemotherapy (nine courses of oxaliplatin/5-fluorouracil/leucovorin (mFOLFOX6)) for locally advanced lower rectal cancer, underwent abdominal perineal resection with lateral lymph node dissection. He had no remarkable medical or medication history, except for diabetes. He had no family history of FAP. His postoperative recovery was uneventful, with the exception of intrapelvic fluid collection which was drained percutaneously. Histological examination of the tumor revealed a well- to moderately differentiated adenocarcinoma (stage II (pT3N0M0) according to the 7th TNM classification of the International Union Against Cancer) [7]. He was scheduled for regular cancer surveillance after surgery with three monthly serum carcinoembryonic antigen (CEA) and serum carbohydrate antigen 19-9 (CA19-9) measurements and six monthly contrast-enhanced computed tomography (CT) evaluations of the thorax, abdomen, and pelvis. Six months postoperatively, CT revealed a solitary mass (lesion 1) in the pelvic mesentery, 20 mm in size (Fig. 1). Serum CEA and CA19-9 levels were normal. Recurrence of rectal cancer was suspected, but could not be confirmed, and thus, close follow-up was chosen. Another CT, performed 3 months later, showed that the mass had grown to be 35 × 30 mm in size (lesion 1), with two new masses measuring 20 × 15 mm (lesion 2) and 15 × 10 mm (lesion 3) forming at the mesentery of the small bowel (Fig. 2). Pelvic magnetic resonance imaging (MRI) on T2-weighted images revealed that the central part of the tumor was hypointense and the peripheral part was hyperintense, which was gradually enhanced with gadolinium contrast MRI (Fig. 3). Positron emission tomography (PET)-CT showed increased fluorodeoxyglucose (FDG) uptake in all tumors (max standardized uptake value: SUV = 3.31 (lesion 1), 2.55 (lesion 2), and 2.22 (lesion 3)) (Fig. 4). From these imaging findings, we excluded the possibilities of other intra-abdominal tumors (gastrointestinal stromal tumors (GIST), malignant lymphoma, etc.). Considering the patient’s medical history, and multiple occurrences of masses, the most probable diagnosis was recurrence of rectal cancer, with a differential diagnosis of desmoid tumors. Upon evaluation at our multidisciplinary team meeting, three tumors were localized and no other distant metastasis was observed, curative resection was chosen for therapeutic diagnosis.

**Fig. 5:**
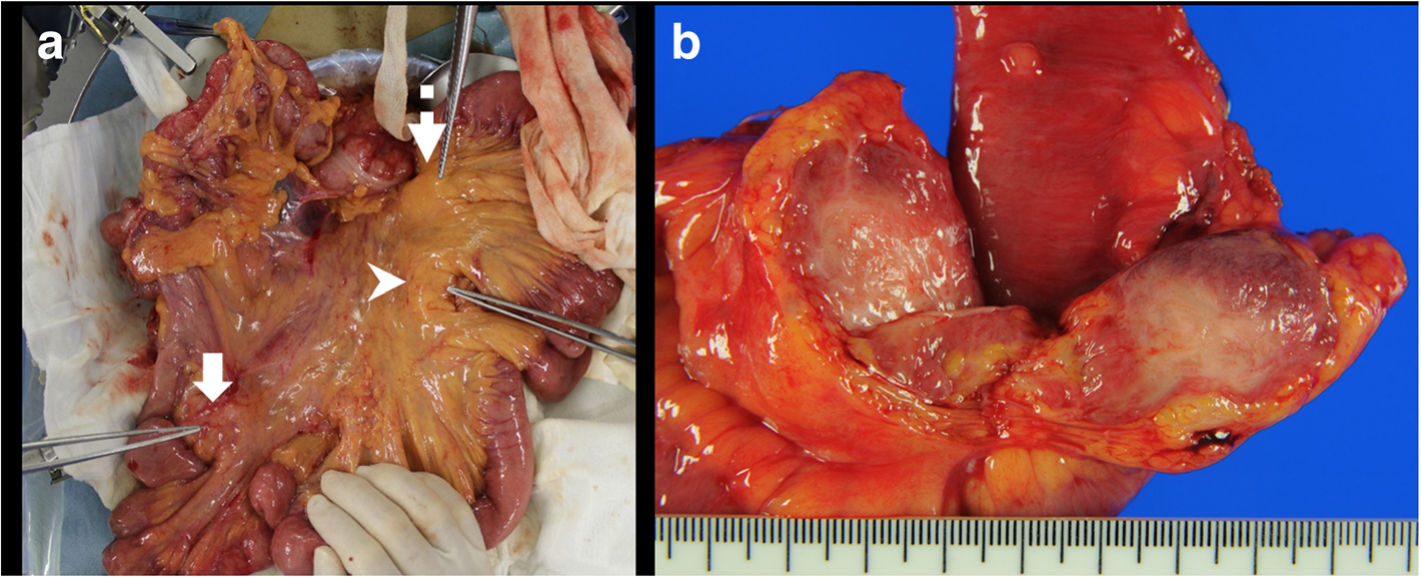
Intraoperative findings and resected specimens. **a** The first tumor (lesion 1, arrow) is located near the terminal ileum, the second (lesion 2, arrowhead) approximately 100 cm proximal to the first lesion, and the third (lesion 3, dotted arrow) near the second tumor. **b** The cut surface of the resected specimen shows smooth, whitish-yellow tissue surrounded by adipose inflammation

**Fig. 6:**
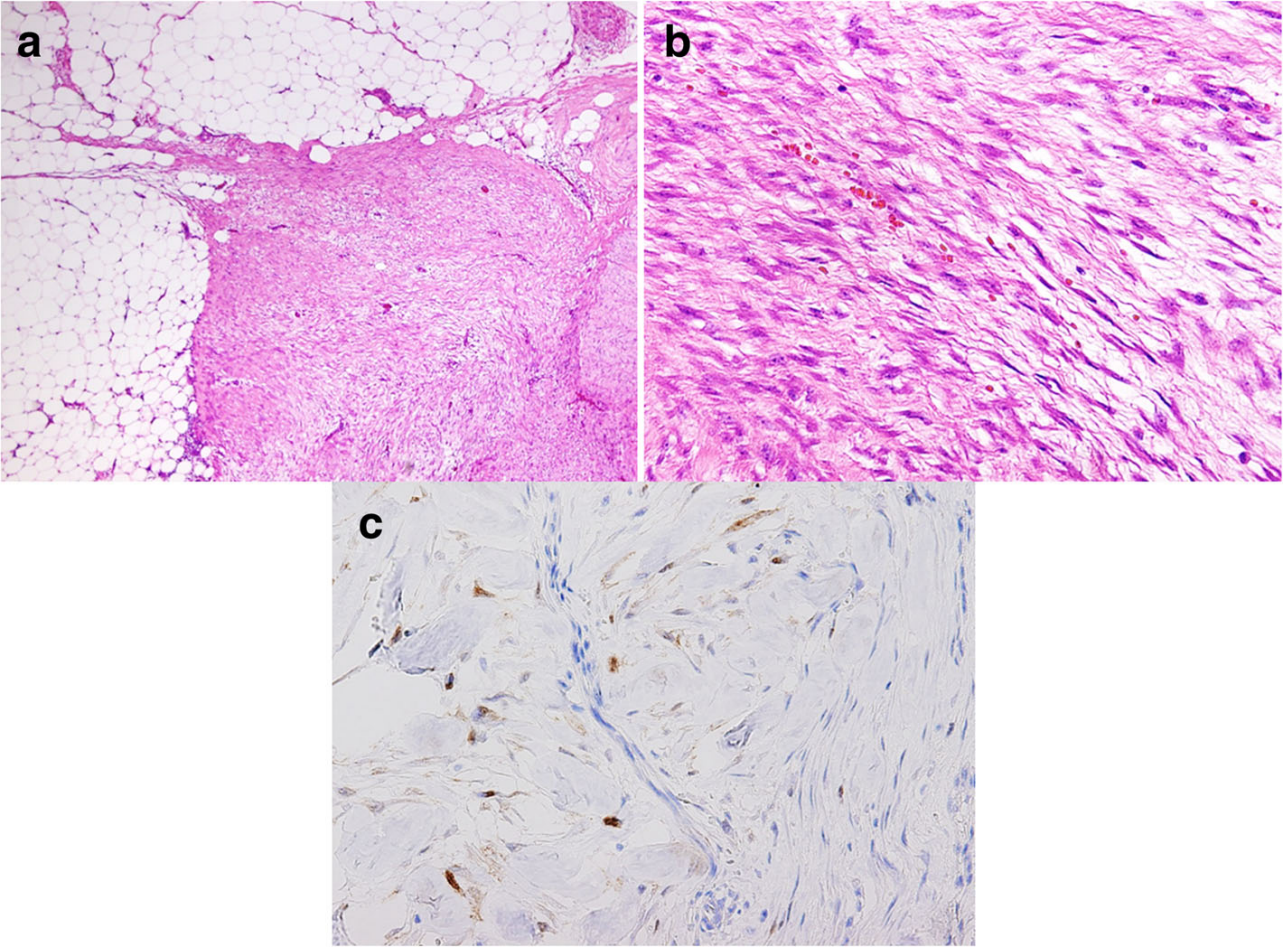
Histological and immunohistological features of desmoid tumors. **a** Hematoxylin and eosin staining shows proliferation of spindle-shaped cells with collagenous stroma. **a** Low-power view. **b** High-power view. **c** Immunohistological staining for β-catenin shows focal staining of cell nuclei

Intraoperatively, three tumors were located at the mesentery of the small bowel. The first tumor (lesion 1) was located near the terminal ileum, the second (lesion 2) approximately 100 cm proximal to the first lesion, and the third (lesion 3) was near the second tumor (Fig. 5a). The most distal tumor was first removed with segmental bowel resection and a specimen was submitted for intraoperative sectioning, which suggested the possibility of desmoid tumors. Since the tumors were growing very rapidly, the remaining two mesenteric tumors were also resected in a similar manner. Macroscopically, the tumors were hard but elastic, with clear borders, measuring 40 × 40 × 30 mm (lesion 1), 40 × 30 × 20 mm (lesion 2), and 30 × 25 × 20 mm (lesion 3). Histological examination revealed the proliferation of spindle-shaped cells and collagenous stroma. No nuclear atypia was observed. The resection margins were negative. Immunohistological examination showed that the nuclei of cells were focally positive for β-catenin (Fig. 6). There was also moderate intratumoral hemorrhage, with no invasion into the adjacent small bowel. The final diagnosis was multiple desmoid tumors, also referred to as desmoid-type fibromatosis. After operation, the patient has not received postoperative adjuvant chemotherapy and remains well 1 year after his last operation without any signs of recurrence of desmoid tumors or rectal cancer.

## Discussion

Mesenteric masses have always been a challenge to evaluate radiologically. CT, MRI, and PET-CT are typically used to differentiate intra-abdominal tumors. On contrast-enhanced CT, mesenteric desmoid tumors appear as a well-marginated and nonspecific, enhancing soft tissue mass that may be iso- or slightly hypodense to surrounding muscle [8]. In our case, CT revealed a well-marginated soft tissue mass, which did not necessarily indicate cancer recurrence. On MRI, the typical appearance of mesenteric fibromatosis is a soft tissue mass with heterogeneous signal intensity on T2-weighted images, the latter of which reflect the variable quantities and distribution of myofibroblasts, extracellular collagen, and myxoid matrix [8]. In our case, the central part of the masses on MRI T2-weighted images was hypointense and the peripheral part was hyperintense. Lesions in our case also exhibited moderate enhancement with gadolinium contrast agents, other than the hypointense collagen bundles which were not enhanced. These findings could be suggestive of desmoid tumors and may be useful to distinguish them from GIST and malignant lymphoma (homogenous enhancement is useful to differentiate fibromatosis from GIST [9]; presenting as a muscular mass is useful to differentiate fibromatosis from lymphoma [8]). But cancer recurrence cannot be denied. On FDG-PET, which enables functional imaging of various tumors, desmoid tumors tend to have low FDG uptake, depending on the amount of cellular tissue and mitotic activity of fibromatosis [10]. In our case, low FDG uptake was seen in all three masses. On these imaging modalities, desmoid tumors and recurrence of rectal cancer were considered; however, due to the patient’s prior history of rectal cancer, recurrence of rectal cancer was considered more plausible.

The pathogenesis of desmoid tumors is a variety of factors, such as FAP, hormonal imbalance (hyperestrogenic states), and antecedent abdominal trauma (including previous surgery). In our case, there was no family history or evidence of polyposis on colonoscopy. Although the patient had received neoadjuvant chemotherapy, pelvic lymph node dissection, and developed postoperative pelvic fluid collection, there are no reports suggesting a direct correlation between desmoid tumors. Hence, in our case, the most probable pathogenesis was surgical trauma.

Remarkable points of our case include very early development after surgical trauma, rapid growth, and multiple occurrences in a non-FAP patient. To the best of our knowledge, there is only one report of desmoid tumors which developed early after surgical trauma and multiple occurrences in a non-FAP patient. Das et al. [6] described a patient who developed multiple desmoid tumors 8 months postoperatively. From a temporal perspective, Shih et al. [11] reported that sporadic postoperative intra-abdominal desmoid tumors can develop 11 months to 7 years after abdominal surgery. In our patient, the desmoid tumor was detected 6 months postoperatively and the new masses were detected 9 months postoperatively. Given the limited number of reports on this condition, the prognosis of desmoid tumors that are found early after surgical trauma and then increase in number is unknown.

A clinically relevant staging system was proposed by the Collaborative Group of the Americans on Inherited Colorectal Cancer (CGA-ICC) in 2005 for the management of FAP patients with intra-abdominal desmoid tumors [12]. According to the staging system, patients who have rapidly growing tumors (> 50% increase in size in 6 months) are classified as stage IV [12]. Prognosis correlates with classification, and the 5-year survival rate for FAP-associated stage IV desmoid tumors is reported to be 76% [3]. Although our case is not FAP-related, it could be regarded as stage IV since tumor size increased from 20 × 20 mm to 40 × 40 × 30 mm in just 6 months. Our patient will require close follow-up to see if this could result in poorer prognosis or earlier recurrence of desmoid tumors.

Desmoid tumors are characterized by unpredictable clinical behavior. For instance, although most grow progressively larger over time, some grow indolent or stay stable and some occasionally spontaneously regress. Since spontaneous regression and stabilization are possible, surgical resection is not always necessary with asymptotic desmoid tumors and an initial “wait-and-see” approach is reasonable in such cases. Indeed, some patients benefit from foregoing aggressive therapies and adopting a “wait-and-see” approach, although the features that identify such patients with a low risk of disease progression are unclear [13]. In contrast, when the recurrence of malignancy is suspected, as in our case, surgical resection to confirm histology is mandatory before systemic chemotherapy, as this could avoid unnecessary chemotherapy. In our case, the recurrence of rectal cancer was still the most probable diagnosis based on the patient’s medical history and multiple occurrences, whereas desmoid tumor was the differential diagnosis. To address peritoneal metastasis of colorectal cancer, Japan has unique therapeutic strategies which differ from those of Western countries. According to Japanese guidelines 2016 for the treatment of colorectal cancer by the Japanese Society for Cancer of the Colon and Rectum [14], complete resection is desirable for metastasis localized to the adjacent peritoneum and complete resection is also considered for limited metastasis to the distant peritoneum when easily resectable. In our case three masses were localized at the mesentery of the small bowel; thus, complete resection was chosen even if these were all peritoneal metastasis. The tumors were finally diagnosed as multiple desmoid tumors, rather than recurrence of rectal cancer, allowing unnecessary systemic chemotherapy to be avoided.

## Conclusions

We describe a rare case of multiple, rapidly growing, mesenteric desmoid tumors that were difficult to distinguish from recurrence of rectal cancer. In patients with a history of surgery for intra-abdominal malignancies, it may be difficult to distinguish the recurrence of malignancy from desmoid tumors. However, as was evident in the present case, the possibility of desmoid tumors should not be excluded even when there are multiple, rapidly growing masses.

## Abbreviations

CA19-9: Carbohydrate antigen 19-9
CEA: Carcinoembryonic antigen
CT: Computed tomography
FAP: Familial adenomatous polyposis
FDG: Fluorodeoxyglucose
GIST: Gastrointestinal stromal tumors
MRI: Magnetic resonance imaging
PET: Positron emission tomography
SUV: Standardized uptake value
